# Supplementary material for: Human responses to the DNA prime/chimpanzee adenovirus (ChAd63) boost vaccine identify CSP, AMA1 and TRAP MHC Class I-restricted epitopes
Source: PLoS One. 2025 Feb 13;20(2):e0318098. doi: 10.1371/journal.pone.0318098 (PMC11825025; doi:10.1371/journal.pone.0318098)
Supplement: S1 Table — (DOCX) [file pone.0318098.s001.docx]

**S1 Table. Cohort CA and Cohort CAT participants: HLA alleles and supertypes**

| **CA Cohort** | | | | | |
| --- | --- | --- | --- | --- | --- |
| **Participant** | **Status** | **HLA-A 1 (ST)** | **HLA-A 2 (ST)** | **HLA-B 1 (ST)** | **HLA-B 2 (ST)** |
| **v01** | NP | A*02:03 (A02) | A*11:01 (A03) | B*13:01 (unclass.) | B*38:02 (unclass.) |
| **v08** | NP | A*02:01 (A02) | A*24:02 (A24) | B*27:05 (B27) | B*35:01 (B07) |
| **v20** | NP | A*02:01 (A02) | A*23:01 (A24) | B*14:01 (B27) | B*45:01 (B44) |
| **v32** | NP | A*03:01 (A03) | A*03:01 (A03) | B*07:02 (B07) | B*07:02 (B07) |
| **v40** | NP | A*30:01 (A01 A03) | A*33:03 (A03) | B*42:01 (B07) | B*57:03 (B58) |
| **v41** | NP | A*30:01 (A01 A03) | A*74:01 (A03) | B*42:01 (B07) | B*44:03 (B44) |
| **v53** | NP | A*11:01 (A03) | A*24:07 (unclass.) | B*13:01 (unclass.) | B*15:02 (B62) |
| **v59** | NP | A*02:01 (A02) | A*02:01 (A02) | B*18:01 (B44) | B*40:02 (B44) |
| **v63** | NP | A*02:01 (A02) | A*11:01 (A03) | B*07:02 (B07) | B*44:02 (B44) |
| **v68** | NP | A*01:01 (A01) | A*03:01 (A03) | B*14:02 (B27) | B*40:02 (B44) |
| **v78** | NP | A*01:01 (A01) | A*02:01 (A02) | B*15:13(B62) | B*44:02 (B44) |
| **v81** | NP | A*02:01 (A02) | A*26:01 (A01) | B*07:02 (B07) | B*38:01 (B27) |
| **v82** | NP | A*24:02 (A24) | A*66:02 (A03) | B*56:01 (B07) | B*58:01 (B58) |
| **v83** | NP | A*23:01 (A24) | A*24:02 (A24) | B*40:01 (B44) | B*44:03 (B44) |
| **v88** | NP | A*02:01 (A02) | A*02:01 (A02) | B*44:02 (B44) | B*51:01 (B07) |
| **v90** | P | A*33:03 (A03) | A*33:03 (A03) | B*44:03 (B44) | B*58:01 (B58) |
|  |  |  |  |  |  |
| **CAT Cohort** | | | | | |
| **Participant** | **Status** | **HLA-A 1 (ST)** | **HLA-A 2 (ST)** | **HLA-B 1 (ST)** | **HLA-B 2 (ST)** |
| **v09** | NP | A*01:01 (A01) | A*23:01 (A24) | B*07:02 (B07) | B*07:05 (B07) |
| **v12** | P | A*02:01 (A02) | A*32:01 (A01) | B*35:01 (B07) | B*44:03 (B44) |
| **v15** | NP | A*23:01 (A24) | A*36:01 (A01) | B*44:03 (B44) | B*53:01 (B07) |
| **v17** | P | A*02:01 (A02) | A*02:01 (A02) | B*08:01 (B08) | B*13:02 (unclass.) |
| **v24** | P | A*02:01 (A02) | A*30:01 (A01 A03) | B*35:01 (B07) | B*42:02 (unclass.) |
| **v33** | NP | A*23:01 (A24) | A*33:03 (A03) | B*51:01 (B07) | B*58:01 (B58) |
| **v34** | NP | A*03:01 (A03) | A*30:02 (A01) | B*14:02 (B27) | B*35:01 (B07) |
| **v35** | NP | A*02:01 (A02) | A*02:06 (A02) | B*39:05 (B27) | B*58:01 (B58) |
| **v46** | P | A*11:01 (A03) | A*68:03 (A03) | B*27:05 (B27) | B*35:17 (B07) |
| **v57** | NP | A*02:01 (A02) | A*26:01 (A01) | B*07:02 (B07) | B*44:02 (B44) |
| **v64** | NP | A*03:01 (A03) | A*68:01 (A03) | B*15:01(B62) | B*81:01 (B07) |
| **v70** | NP | A*02:01 (A02) | A*24:02 (A24) | B*27:05 (B27) | B*55:01 (B07) |
| **v73** | NP | A*02:01 (A02) | A*26:01 (A01) | B*35:01 (B07) | B*40:01 (B44) |
| **v74** | P | A*03:01 (A03) | A*29:02 (A01 A24) | B*15:01 (B62) | B*38:01 (B27) |
| **v75** | NP | A*02:01 (A02) | A*11:01 (A03) | B*35:03 (B07) | B*51:01 (B07) |
| **v76** | NP | A*01:01 (A01) | A*30:02 (A01) | B*08:01 (B08) | B*18:01 (B44) |

Each subject was HLA typed (Methods) and HLA alleles were grouped into supertypes (ST) or remained unclassified (unclass.). Participants were protected (P) or non-protected (NP).
