# Supplementary material for: Human responses to the DNA prime/chimpanzee adenovirus (ChAd63) boost vaccine identify CSP, AMA1 and TRAP MHC Class I-restricted epitopes
Source: PLoS One. 2025 Feb 13;20(2):e0318098. doi: 10.1371/journal.pone.0318098 (PMC11825025; doi:10.1371/journal.pone.0318098)
Supplement: S2 Table — (DOCX) [file pone.0318098.s002.docx]

**S2 Table. Cohort CA: FluoroSpot IFN-γ and GzB responses for protected participant v90 (HLA A03/A03, B44/B58) to AMA1 Ap8, and Ap10 peptide sub pools, single 15mer peptides, and synthesized predicted minimal epitopes**

| **A. Response to sub pools and 15mer components** | | | | |  | **B. Response to positive 15mers and predicted epitopes** | | | |
| --- | --- | --- | --- | --- | --- | --- | --- | --- | --- |
| **Pool/**  **15mer** | **15mer Sequence** | **IFN-γ**  **sfc/m** | **GzB**  **sfc/m** | **HLA**  **Restriction/ST of predicted epitope** |  | **15mer Sequence** | **predicted Epitope** | **IFN-γ**  **sfc/m** | **GzB**  **sfc/m** |
| **Ap8** |  | **418** | **175** |  |  | **A97** |  |  |  |
| A92 | EGFKNKNASMIKSAF | 8 | 25 |  |  | FKADRYKSHGKGYNW |  | **753** | **393** |
| A93 | NKNASMIKSAFLPTG | 0 | 5 |  |  | FKAD**(RYKSHGKGY)**NW | **RYKSHGKGY** | 10 | 20 |
| A94 | SMIKSAFLPTGAFKA | 3 | 0 |  |  | FKA**(DRYKSHGKGY)**NW | **DRYKSHGKGY** | 0 | 53 |
| A95 | SAFLPTGAFKADRYK | 3 | 0 |  |  | FKADR**(YKSHGKGYNW)** | **YKSHGKGYNW** | **263** | 87 |
| A96 | PTGAFKADRYKSHGK | 0 | 0 |  |  | **(FKADRYKSH)**GKGYNW | **FKADRYKSH** | 3 | 0 |
| **A97** | **(FKADRYKSHGKGYNW)** | **585** | **95** | **B*58:01 (B58)** |  | F**(KADRYKSHGK)**GYNW | **KADRYKSHGK** | 7 | 0 |
| **A98** | **(RYKSHGKGYNW)**GNYN | **110** | 5 | **B*58:01 (B58)** |  | FKADRY**(KSHGKGYNW)** | **KSHGKGYNW** | **467** | 87 |
| A99 | HGKGYNWGNYNTETQ | 0 | 0 |  |  | FKA**(DRYKSHGK)**GYNW | **DRYKSHGK** | 0 | 0 |
| A100 | YNWGNYNTETQKCEI | 0 | 10 |  |  | FK**(ADRYKSHGK)**GYNW | **ADRYKSHGK** | 3 | 0 |
| A101 | NYNTETQKCEIFNVK | 3 | 13 |  |  |  |  |  |  |
| A102 | ETQKCEIFNVKPTCL | 0 | 15 |  |  |  |  |  |  |
| **A103** | **(CEIFNVKPT)**CLINNS | 3 | **65** | **B*45:01 (B44)** |  | **A126** |  |  |  |
| A104 | NVKPTCLINNSSYIA | 3 | 0 |  |  | TCRFFVCKCVERRAE |  | **107** | 63 |
| **Ap10** |  | 0 | 0 |  |  | TC**(RFFVCKCVER)**RAE | **RFFVCKCVER** | **180** | **123** |
| A126 | TC**(RFFVCKCVER)**RAE | 48 | 0 | **A*33:03 (A03)** |  |  |  |  |  |
| **Ap11** |  |  |  |  |  |  |  |  |  |
| A136 | TYDKMK**(IIIASSAAV)** | 15 | 0 | **A*02:01** |  | **A136** | **IIIASSAAV** | 7 | 0 |
| A137 | MK**(IIIASSAAVAV)**LA | 0 | 3 | **B*58:01** |  |  |  |  |  |

PBMCs were collected from the participant post-ChAd63/pre-CHMI. **(A)** All 15mer peptides within Ap8 were tested in FluoroSpot assays. Positive activities for 15mers A97, A98, and A103 are shown in bold and predicted epitopes within the15mers are shown in bold with parenthesis and underlined. **(B)** Predicted minimal epitopes within A97 and A126 shown were synthesized and tested. Positive activities are shown in bold.
