# Supplementary material for: Human responses to the DNA prime/chimpanzee adenovirus (ChAd63) boost vaccine identify CSP, AMA1 and TRAP MHC Class I-restricted epitopes
Source: PLoS One. 2025 Feb 13;20(2):e0318098. doi: 10.1371/journal.pone.0318098 (PMC11825025; doi:10.1371/journal.pone.0318098)
Supplement: S3 Table — (DOCX) [file pone.0318098.s003.docx]

**S3 Table. Cohort CA:** **FluoroSpot IFN-γ and GzB responses for non-protected participant v32 (HLA A03/A03, B07/B07) to AMA1 Ap8 and Ap10 subpools, 15mer peptides, and synthesized predicted epitopes**

| **A. Response to sub pools and 15mer components** | | | | |  | **B. Response to positive 15mers and predicted epitopes** | | | |
| --- | --- | --- | --- | --- | --- | --- | --- | --- | --- |
| **Pool/**  **15mer** | **15mer Sequence** | **IFN-γ**  **sfc/m** | **GzB**  **sfc/m** | **HLA**  **Restriction/ST of predicted epitope** |  | **15mer Sequence** | **Epitope** | **IFN-γ**  **sfc/m** | **GzB**  **sfc/m** |
| **Ap8** |  | 35 | 0 |  |  | **A94** |  |  |  |
| A92 | EGFKNKNASMIKSAF | 1 | 8 |  |  | SMIKSAFLPTGAFKA |  | **45** | 0 |
| A93 | NKNASMIKSAFLPTG | 0 | 0 |  |  | SMIKSA**(FLPTGAFKA)** | **FLPTGAFKA** | 8 | 0 |
| **A94** | **(SMIKSAFLPTGAFKA)** | 35 | 35 |  |  | SMIK**(SAFLPTGAFK)**A | **SAFLPTGAFK** | **105** | 0 |
| **A95** | **(SAFLPTGAFKA)**DRYK | **65** | 0 | **A*03:01 (A03)** |  | **(SMIKSAFLPT)**GAFKA | **SMIKSAFLPT** | 13 | 0 |
| A96 | PTGAFKADRYKSHGK | 1 | 0 |  |  | SMIK**(SAFLPTGAF)**KA | **SAFLPTGAF** | 5 | 0 |
| A97 | FKADRYKSHGKGYNW | 3 | 0 |  |  |  |  |  |  |
| A98 | RYKSHGKGYNWGNYN | 1 | 3 |  |  | **A95** |  |  |  |
| A99 | HGKGYNWGNYNTETQ | 0 | 0 |  |  | SAFLPTGAFKADRYK |  | **75** | 0 |
| A100 | YNWGNYNTETQKCEI | 10 | 3 |  |  | SA**(FLPTGAFKA)**DRYK | **FLPTGAFKA** | 8 | 0 |
| A101 | NYNTETQKCEIFNVK | 0 | 8 |  |  | **(SAFLPTGAFK**)ADRYK | **SAFLPTGAFK** | **105** | 0 |
| A102 | ETQKCEIFNVKPTCL | 4 | 0 |  |  | **(SAFLPTGAF)**KADRYK | **SAFLPTGAF** | 5 | 0 |
| A103 | CEIFNVKPTCLINNS | 0 | 3 |  |  |  |  |  |  |
| A104 | NVKPTCLINNSSYIA | 0 | 0 |  |  |  |  |  |  |
| **Ap10** |  | **735** | 48 |  |  |  |  |  |  |
| A118 | EGNKKIIAPRIFISD | 0 | 10 |  |  |  |  |  |  |
| A119 | KIIAPRIFISDDKDS | 1 | 0 |  |  | **A125** |  |  |  |
| A120 | PRIFISDDKDSLKCP | 0 | 0 |  |  | VSNSTCRFFVCKCVE |  | **1015** | **183** |
| A121 | ISDDKDSLKCPCDPE | 4 | 0 |  |  | VS(**NSTCRFFVCK)**CVE | **NSTCRFFVCK** | **718** | **113** |
| A122 | KDSLKCPCDPEMVSN | 4 | 0 |  |  | VSN**(STCRFFVCK)**CVE | **STCRFFVCK** | **1418** | **405** |
| A123 | KCPCDPEMVSNSTCR | 1 | 0 |  |  | **(VSNSTCRFF)**VCKCVE | **VSNSTCRFF** | 0 | 0 |
| A124 | DPEMVSNSTCRFFVC | 0 | 0 |  |  |  |  |  |  |
| **A125** | **(VSNSTCRFFVCK)**CVE | **580** | 8 | **A*03:01 (A03)** |  | **A126** |  |  |  |
| **A126** | TC(**RFFVCKCVER)**RAE | **108** | 3 | **A*03:01 (A03)** |  | TCRFFVCKCVERRAE |  | **88** | 0 |
| A127 | FVCKCVERRAEVTSN | 11 | 3 |  |  | TC(**RFFVCKCVER)**RAE | **RFFVCKCVER** | 13 | 0 |
| A128 | CVERRAEVTSNNEVV | 1 | 0 |  |  |  | | | |
| A129 | RAEVTSNNEVVVKEE | 0 | 8 |  |  |  |  |  |  |
| A130 | TSNNEVVVKEEYKDE | 13 | 3 |  |  |  |  |  |  |

PBMCs were collected from the participant post-ChAd63/pre-CHMI. **(A)** All 15mer peptides within Ap8 and Ap10 were tested in FluoroSpot assays. Positive activities are shown in bold. Predicted epitopes within positive 15mers, A95, A125, A126 and not positive 15mer A94 are shown in bold with parenthesis and underlined. **(B)** Predicted minimal epitopes shown were synthesized and tested. Positive activities are shown in bold.
