# Supplementary material for: Human responses to the DNA prime/chimpanzee adenovirus (ChAd63) boost vaccine identify CSP, AMA1 and TRAP MHC Class I-restricted epitopes
Source: PLoS One. 2025 Feb 13;20(2):e0318098. doi: 10.1371/journal.pone.0318098 (PMC11825025; doi:10.1371/journal.pone.0318098)
Supplement: S4 Table — (DOCX) [file pone.0318098.s004.docx]

**S4 Table. Cohort CAT: FluoroSpot IFN-γ and GzB responses for protected participant v12 (HLA A02/A01, B07/B44) to AMA1 subpool Ap8, 15mer peptides, and synthesized predicted epitopes**

| **A. Response to sub pool and 15mers components** | | | | |  | **B. Response to positive 15mer and predicted epitopes** | | | |
| --- | --- | --- | --- | --- | --- | --- | --- | --- | --- |
| **Pool/15mer** | **15mer Sequence** | **IFN-γ**  **sfc/m** | **GzB**  **sfc/m** | **HLA**  **Restriction/ST of predicted epitope** |  | **15mer Sequence** | **Minimal Epitope** | **IFN-γ**  **sfc/m** | **GzB**  **sfc/m** |
| **Ap8** |  | **158** | 0 |  |  |  |  |  |  |
| A92 | EGFKNKNASMIKSAF | 1 | 14 |  |  | **A97** | | |  |
| A93 | NKNASMIKSAFLPTG | 0 | 1 |  |  |  |  |  |  |
| A94 | SMIKSAFLPTGAFKA | 1 | 8 |  |  |  |  |  |  |
| A95 | SAFLPTGAFKADRYK | 0 | 0 |  |  |  |  |  |  |
| A96 | PTGAFKADRYKSHGK | 0 | 0 |  |  |  |  |  |  |
| A97 | **(FKADRYKSHGKGYNW)** | **188** | 15 | **A*32:01 (A01)** |  | FKADRYKSHGKGYNW |  | **353** | 68 |
| A98 | RYKSHGKGYNWGNYN | 0 | 0 |  |  | FKAD**(RYKSHGKGY)**NW | **RYKSHGKGY** | 15 | 28 |
| A99 | HGKGYNWGNYNTETQ | 0 | 0 |  |  | FKA**(DRYKSHGKGY)**NW | **DRYKSHGKGY** | **40** | 70 |
| A100 | YNWGNYNTETQKCEI | 0 | 0 |  |  | FKADR(**YKSHGKGYNW)** | **YKSHGKGYNW** | **168** | 135 |
| A101 | NYNTETQKCEIFNVK | 3 | 0 |  |  | **(FKADRYKSH)**GKGYNW | **FKADRYKSH** | 25 | 65 |
| A102 | ETQKCEIFNVKPTCL | 46 | 21 |  |  | F**(KADRYKSHGK)**GYNW | **KADRYKSHGK** | 15 | 75 |
| A103 | CEIFNVKPTCLINNS | 0 | 36 |  |  | FKADRY(**KSHGKGYNW)** | **KSHGKGYNW** | **88** | 50 |
| A104 | NVKPTCLINNSSYIA | 0 | 0 |  |  | FKA**(DRYKSHGK)**GYNW | **DRYKSHGK** | **113** | 115 |
|  | | | | |  | FK**(ADRYKSHGK)**GYNW | **ADRYKSHGK** | 0 | 38 |

PBMCs were collected from the participant post-ChAd63/pre-CHMI. **(A)** All 15mer peptides within Ap8 were tested in FluoroSpot assays. Positive activities for A97 are shown in bold. **(B)** Predicted epitopes within positive 15mer A97 is shown in bold with parenthesis and underlined. Predicted minimal epitopes shown were synthesized and tested. Positive activities are shown in bold.
