# Supplementary material for: Human responses to the DNA prime/chimpanzee adenovirus (ChAd63) boost vaccine identify CSP, AMA1 and TRAP MHC Class I-restricted epitopes
Source: PLoS One. 2025 Feb 13;20(2):e0318098. doi: 10.1371/journal.pone.0318098 (PMC11825025; doi:10.1371/journal.pone.0318098)
Supplement: S5 Table — (DOCX) [file pone.0318098.s005.docx]

**S5 Table. Cohort CAT: FluoroSpot IFN-γ and GzB responses for protected participant v12 (HLA A02/A01, B07/B44) to CSP subpool Cp5 and 15mer peptides**

| **Response to sub pools and 15mer components** | | | | |
| --- | --- | --- | --- | --- |
| **Pool/**  **15mer** | **15mer Sequence** | **IFN-γ**  **sfc/m** | **GzB**  **sfc/m** | **HLA Restriction/ST of predicted epitope** |
| **Cp5** |  | **88** | 6 |  |
| C37 | NKNNQGNGQGHNMPN | 1 | 0 |  |
| C38 | QGNGQGHNMPNDPNR | 0 | 0 |  |
| C39 | QGHNMPNDPNRNVDE | 0 | 0 |  |
| C40 | MPNDPNRNVDENANA | 0 | 0 |  |
| C41 | PNRNVDENANANSAV | 0 | 0 |  |
| C42 | VDENANANSAVKNNN | 1 | 0 |  |
| C43 | ANANSAVKNNNNEEP | 0 | 0 |  |
| C44 | SAVKNNNNEEPSDKH | 0 | 0 |  |
| C45 | NNNNE**(EPSDKHIKEY)** | **95** | 26 | **B*35:01 (B07)** |
|  | NNNNEE**(PSDKHIKEY)** | **95** | 26 | **A*01:01 (A01)** |

PBMCs were collected from the participant post-ChAd63/pre-CHMI. All 15mer peptides within Cp5 were tested in FluoroSpot assays. Positive activities are shown in bold. Predicted epitopes within positive 15mers are shown in bold with parenthesis and underlined.
