# Supplementary material for: Human responses to the DNA prime/chimpanzee adenovirus (ChAd63) boost vaccine identify CSP, AMA1 and TRAP MHC Class I-restricted epitopes
Source: PLoS One. 2025 Feb 13;20(2):e0318098. doi: 10.1371/journal.pone.0318098 (PMC11825025; doi:10.1371/journal.pone.0318098)
Supplement: S6 Table — (DOCX) [file pone.0318098.s006.docx]

**S6 Table. Cohort CAT: FluoroSpot IFN-γ and GzB responses for protected participant v12 (HLA A02/A01, B07/B44) to 3D7 TRAP TD1 and TD2 sub pools and positive 15mer peptides containing predicted epitopes**

| **A. Response to subpools and 15mer components** | | | | |  | **B. Response to subpools and 15mer components** | | | | |
| --- | --- | --- | --- | --- | --- | --- | --- | --- | --- | --- |
| **Pool/**  **15mer #** | **15mer Sequence** | **IFN-γ**  **sfc/m** | **GzB**  **sfc/m** | **HLA**  **Restriction/ST of predicted epitope** |  | **Pool/**  **15mer #** | **15mer Sequence** | **IFN-γ**  **sfc/m** | **GzB**  **sfc/m** | **HLA**  **restriction** |
| **TD1** |  | **105** | 20 |  |  | **TD2** |  | **175** | **55** |  |
| SS-1 | MN**(HLGNVKYLV)**IVFL | **33** | **55** | **A*02:01 (A02)** |  | SS-26 | IRLHSDASKNKEKAL | 0 | 15 |  |
| SS-2 | GNV**(KYLVIVFLI)**FFD | 23 | **35** | **A*32:01 (A01)** |  | SS-27 | SDASKNKEKALIIIK | 0 | 20 |  |
| SS-3 | YLVIVFLIFFDLFLV | 10 | 0 |  |  | SS-28 | KNKEK**(ALIIIKSL)**LS | **38** | **55** | **A*02:01 (A02)** |
| SS-4 | V**(FLIFFDLFLV)**NGRD | 18 | **30** | **A*02:01 (A02)** |  | SS-29 | KALIIIKSLLSTNLP | 0 | 0 |  |
| SS-5 | FFDL**(FLVNGRDVQ)**NN | 25 | **65** | **B*35:01 (B07)** |  | SS-30 | IIK**(SLLSTNLP)**YGKT | **30** | **65** | **B*35:01 (B07)** |
| 55-6 | **(FLVNGRDVQ)**NNIVDE | 8 | 5 |  |  | SS-31 | LLSTNLPYGKTNLTD | 0 | 20 |  |
| SS-7 | GRDVQNNIVDEIKYR | 3 | 0 |  |  | SS-32 | NLPYG**(KTNLTDALL)**Q | **30** | 25 | **A*32:01 (A01)** |
| SS-8 | QNNIV**(DEIKYREEV)**C | **43** | **53** | **B*44:03 (B44)** |  | SS-33 | G**(KTNLTDALL)**QVRKH | 0 | 8 |  |
| SS-9 | V**(DEIKYREEVC)**NDEV | **33** | **100** | **B*44:03 (B44)** |  | SS-34 | LTDALLQVRKHLNDR | 0 | 15 |  |
| SS-10 | KYR**(EEVCNDEVDL)**YL | 10 | **35** | **B*44:03 (B44)** |  | SS-35 | LLQVRKHLNDRINRE | 0 | 10 |  |
| SS-11 | E**(VCNDEVDLY)**LLMDC | 25 | **75** | **B*35:01 (B07)** |  | SS-36 | RK**(HLNDRINRE)**NANQ | 3 | **43** | **A*02:01 (A02)** |
| SS-12 | DEVDLY**(LLMDCSGSI)** | 10 | **40** | **A*02:01 (A02)** |  | SS-37 | NDRIN**(RENANQLVV)**I | 0 | 25 |  |
| SS-13 | LY**(LLMDCSGSI)**RRHN | 5 | 23 |  |  | SS-38 | N**(RENANQLVV)**ILTDG | **200** | **70** | **B*44:03 (B44)** |
|  |  |  |  |  |  |  | NRE**(NANQLVVIL)**TDG | **200** | **70** | **B*35:01 (B07)** |
| SS-14 | MDCS**(GSIRRHNW)**VNH | 25 | **53** | **A*32:01 (A01)** |  | SS-39 | ANQLVVILTDGIPDS | 0 | 23 |  |
| SS-15 | GSIR**(RHNWVNHAV)**PL | 18 | **58** | **A*32:01 (A01)** |  | SS-40 | VVILTDGIPDSIQDS | 0 | 25 |  |
| SS-16 | RHNWVN**(HAVPLAMKL)** | 5 | **48** | **B*35:01 (B07)** |  | SS-41 | TDGIPDSIQDSLKES | 0 | 18 |  |
| SS-17 | VN**(HAVPLAMK)**LIQQL | 15 | **63** | **B*35:01 (B07)** |  | SS-42 | P**(DSIQDSLKE)**SRKLS | 8 | **38** | **B*35:01 (B07)** |
| SS-18 | VP**(LAMKLIQQL)**NLND | 3 | **28** | **B*35:01 (B07)** |  | SS-43 | QDSL**(KESRKLSDRG)**V | **28** | **53** | **B*44:03 (B44)** |
| SS-19 | MKLIQQLNLNDNAIH | 0 | 18 |  |  | SS-44 | KESR**(KLSDRGVKI)**AV | 0 | **38** | **A*32:01 (A01)** |
| SS-20 | QQL**(NLNDNAIHL)**YAS | 15 | **40** | **A*02:01 (A02)** |  | SS-45 | **(KLSDRGVKI)**AVFGIG | 3 | 10 |  |
| SS-21 | LND**(NAIHLYASVF)**SN | **73** | **70** | **B*35:01 (B07)** |  | SS-46 | RGVKI**(AVFGIGQGI)**N | 10 | **65** | **A*32:01 (A01)** |
| SS-22 | **(AIHLYASVF)**SNNARE | **80** | **125** | **B*35:01 (B07)** |  | SS-47 | I**(AVFGIGQGIN)**VAFN | 15 | 25 |  |
| SS-23 | **(YASVFSNNA)**REIIRL | 13 | **40** | **B*35:01 (B07)** |  | SS-48 | GIGQGINVAFNRFLV | 3 | 10 |  |
| SS-24 | FSNN**(AREIIRLHSDA)** | 0 | **38** | **B*44:03 (B44)** |  | SS-49 | GINVAFNRFLVGCHP | 0 | 8 |  |
| **SS-25** | **(AREIIRLHSDA)**SKNK | 0 | 0 |  |  | **SS-50** | AFNRFLVGCHPSDGK | 0 | 13 |  |

PBMCs were collected from the participant post-ChAd63/pre-CHMI. (**A)** All 15mer peptides within TD1, and **(B)** TD2 were tested in FluoroSpot assays. Positive activities are shown in bold and predicted minimal epitopes within 15mers are shown in bold within parenthesis and underlined. Amino acids in the predicted epitopes that vary between 3D7 and T9/96 are shown in red.
