# Supplementary material for: Human responses to the DNA prime/chimpanzee adenovirus (ChAd63) boost vaccine identify CSP, AMA1 and TRAP MHC Class I-restricted epitopes
Source: PLoS One. 2025 Feb 13;20(2):e0318098. doi: 10.1371/journal.pone.0318098 (PMC11825025; doi:10.1371/journal.pone.0318098)
Supplement: S7 Table — (DOCX) [file pone.0318098.s007.docx]

**S7 Table. Cohort CAT: FluoroSpot** **IFN-γ and GzB responses for protected participant v24 (HLA A02/A01A03, B07/B42 [unclassified]) to AMA1 Ap1, Ap3 and Ap9 subpools, 15mer peptides, and synthesized predicted epitopes**

| **A. Response to sub pools and 15mer components** | | | | |  | **B. Response to sub pools and 15mer components** | | | | |
| --- | --- | --- | --- | --- | --- | --- | --- | --- | --- | --- |
| **Pool/**  **15mer** | **15mer Sequence** | **IFN-γ**  **sfc/m** | **GzB**  **sfc/m** | **HLA**  **Restriction/ST of predicted epitope** |  | **Pool/**  **15mer** | **15mer Sequence** | **IFN-γ**  **sfc/m** | **GzB**  **sfc/m** | **HLA**  **Restriction/ST of predicted epitope** |
| **Ap1** |  | **298** | 0 |  |  | **Ap3** |  | 38 | 0 |  |
| A1 | MRKLYCVLLLSAFEF | 1 | 0 |  |  | A27 | YMGNPWTEYMAKYDI | 5 | 0 |  |
| A2 | YCVLLLSAFEFTYMI | 0 | 0 |  |  | A28 | PWTEYMAKYDIEEVH | 3 | 0 |  |
| A2 | LLSAFEFTYMINFGR | 1 | 0 |  |  | A29 | YMAKYDIEEVHGSGI | 1 | 0 |  |
| A4 | FEFTYMINFGRGQNY | 9 | 0 |  |  | A30 | YDIEEVHGSGIRVDL | 0 | 0 |  |
| A5 | YMINFGRGQNYWEHP | 1 | 0 |  |  | A31 | EVHGSGIRVDLGEDA | 1 | 0 |  |
| A6 | FGRGQNYWEHPYQNS | 18 | 0 |  |  | A32 | SGIRVDLGEDAEVAG | 0 | 0 |  |
| A7 | Ac-QNYWE**(HPYQNSDVY)**R | **175** | 0 | **B*35:01 (B07)** |  | A33 | VDLGEDAEVAGTQYR | 0 | 0 |  |
| A8 | E**(HPYQNSDVY)**RPINE | **153** | 0 | **B*35:01 (B07)** |  | A34 | EDAEVAGTQYRLPSG | 0 | 0 |  |
| A9 | Ac-QNSDVYRPINEHREH | 0 | 0 |  |  | A35 | VAGTQYRLPSGKCPV | 0 | 0 |  |
| A10 | VYRPINEHREHPKEY | 0 | 0 |  |  | A36 | QYR**(LPSGKCPVF)**GKG | **115** | 0 | **B*35:01 (B07)** |
| A11 | INEHRE**(HPKEYEYPL)** | **160** | 19 | **B*42:01 (uncl.)** |  | A37 | PSGKCPVFGKGIIIE | 0 | 0 |  |
| A12 | RE**(HPKEYEYPL)**HQEH | **130** | 0 | **B*42:01 (uncl.)** |  | A38 | CPVFGKGIIIENSNT | 0 | 0 |  |
| A13 | KEYEYPLHQEHTYQQ | 4 | 0 |  |  | A39 | GKG**(IIIENSNTTF)**LT | **100** | 71 | **B*35:01 (B07)** |
| **Ap9** |  | **418** | 0 |  |  | **C. Response to positive 15mers and predicted epitopes** | | | |  |
| A105 | TCLINNSSYIATTAL | 0 | 0 |  |  |  |  |  |  |  |
| A106 | NNSSYIATTALSHPI | 0 | 0 |  |  | A11 | INEHRE**(HPKEYEYPL)** | **400** | **43** |  |
| A107 | YIATTALSHPIEVEN | 10 | 0 |  |  | A12 | RE**(HPKEYEYPL)**HQEH | **340** | **83** |  |
| A108 | TALS**(HPIEVENNF)**PC | **515** | 0 | **B*35:01 (B07)** |  |  | **HPKEYEYPL** | **293** | **40** |  |
| A109 | **(HPIEVENN)**FPCSLYK | **238** | 0 | **B*35:01 (B07)** |  |  |  |  |  |  |
| A110 | VENNFPCSLYKDEIM | 4 | 0 |  |  | A36 | QYR**(LPSGKCPVF)**GKG | **223** | **43** |  |
| A111 | FPCSLYKDEIMKEIE | 9 | 0 |  |  |  | **LPSGKCPVF** | **310** | **77** |  |
| A112 | LYKDEIMKEIERESK | 5 | 0 |  |  | A39 | GKG**(IIIENSNTTF)**LT | 3 | 3 |  |
| A113 | EIMKEIERESKRIKL | 1 | 0 |  |  |  | **IIENSNTTF** | 10 | 17 |  |
| A114 | EIERESKRIKLNDND | 0 | 0 |  |  |  | **IIIENSNTTF** | 3 | 3 |  |
| A115 | ESKRIKLNDNDDEGN | 0 | 0 |  |  |  |  |  |  |  |
| A116 | IKLNDNDDEGNKKII | 0 | 0 |  |  | A108 | TALS**(HPIEVENNF)**PC | **1017** | **103** |  |
| A117 | DNDDEGNKKIIAPRI | 0 | 0 |  |  |  | **HPIEVENNF** | **687** | **140** |  |

PBMCs were collected post-ChAd63/pre-CHMI. (**A**) and **(B)** All 15mer peptides within Ap1, Ap3 and Ap9 were tested in FluoroSpot assays. Positive 15mers activities are shown in bold and predicted minimal epitopes within 15mers are shown in bold with parenthesis and underlined. **(C)** Predicted minimal epitopes within the positive 15mers A11, A12, A36, A39 and A108 were synthesized, and tested. Positive activities are shown in bold.
