# Supplementary material for: Human responses to the DNA prime/chimpanzee adenovirus (ChAd63) boost vaccine identify CSP, AMA1 and TRAP MHC Class I-restricted epitopes
Source: PLoS One. 2025 Feb 13;20(2):e0318098. doi: 10.1371/journal.pone.0318098 (PMC11825025; doi:10.1371/journal.pone.0318098)
Supplement: S9 Table — (DOCX) [file pone.0318098.s009.docx]

**S9 Table. Cohort CAT: FluoroSpot IFN-γ and GzB responses for non-protected participant v33 (HLA A24/A03, B07/B58) to AMA1 Ap4, Ap 8 and Ap 10 subpools,15mer peptides, and synthesized predicted epitopes**

| **A. Response to sub pools and 15mer** | | | | | **B. Response to subpools and 15mer components** | | | | |
| --- | --- | --- | --- | --- | --- | --- | --- | --- | --- |
| **Pool/**  **15mer** | **15mer Sequence** | **IFN-γ**  **sfc/m** | **GzB**  **sfc/m** | **HLA**  **Restriction/ST of predicted epitope** | **Pool/**  **15mer** | **15mer Sequence** | **IFN-γ**  **sfc/m** | **GzB**  **sfc/m** | **HLA**  **Restriction/ST of predicted epitope** |
| **Ap4** |  | **110** | 6 |  | **Ap8** |  | **973** | **170** |  |
| A40 | IIENSNTTFLTPVAT | 4 | 21 |  | A92 | EGFKNKNASMIKSAF | 5 | 13 |  |
| A41 | SNTTFLTPVATGNQY | 0 | 0 |  | A93 | NKNASMIKSAFLPTG | 1 | 0 |  |
| A42 | FLTPVATGNQYLKDG | 1 | 0 |  | A94 | SMIKSAFLPTGAFKA | 1 | 3 |  |
| A43 | VATGNQYLKDGGFAF | 1 | 1 |  | A95 | SAFLPTGAFKADRYK | 0 | 1 |  |
| A44 | NQYLKDGGFAFPPTE | 4 | 0 |  | A96 | PTGAFKADRYKSHGK | 0 | 0 |  |
| A45 | KDGGFAFPPTEPLMS | 0 | 25 |  | A97 | FKADRY**(KSHGKGYNW)** | **1123** | **130** | **B*58:01 (B58)** |
| A46 | FAFPPTEPLMSPMTL | 1 | 0 |  | A98 | RYKS**(HGKGYNWGN)**YN | **395** | 4 | **B*35:01 (B07)** |
| A47 | PTEPLMSPMTLDEMR | 0 | 9 |  | A99 | HGKGYNWGNYNTETQ | 0 | 1 |  |
| A48 | LMSPMTLDEMRHFYK | 4 | 0 |  | A100 | YNWGNYNTETQKCEI | 4 | 0 |  |
| A49 | MTLDEMRHFYKDNKY | 0 | 24 |  | A101 | NYNTETQKCEIFNVK | 0 | 1 |  |
| A50 | E**(MRHFYKDNK)Y**VKNL | **145** | **168** | **A*33:03 (A03)** | A102 | ETQKCEIFNVKPTCL | 0 | 0 |  |
| A51 | FYKDNKYVKNLDELT | **58** | 14 |  | A103 | CEIFNVKPTCLINNS | 1 | 0 |  |
| A52 | NKYVKNLDELTLCSR | 3 | 38 |  | A104 | NVKPTCLINNSSYIA | 0 | 0 |  |
| **Ap10** |  | **68** | 0 |  | **C. Response to positive 15mer and predicted epitopes** | | | |  |
| A118 | EGNKKIIAPRIFISD | 4 | 9 |  | **A50** |  |  |  |  |
| A119 | KIIAPRIFISDDKDS | 4 | 0 |  | EMRHFYKDNKYVKNL |  | **188** | **123** |  |
| A120 | PRIFISDDKDSLKCP | 1 | 0 |  | E**(MRHFYKDNKY)**VKNL | **MRHFYKDNKY** | 0 | 0 |  |
| A121 | ISDDKDSLKCPCDPE | 0 | 0 |  |  |  |  |  |  |
| A122 | KDSLKCPCDPEMVSN | 0 | 0 |  | **A97** |  |  |  |  |
| A123 | KCPCDPEMVSNSTCR | 1 | 1 |  | FKADRYKSHGKGYNW |  | **1230** | **393** |  |
| A124 | DPEMVSNSTCRFFVC | 0 | 0 |  | FKAD**(RYKSHGKGY**)NW | **RYKSHGKGY** | 0 | 3 |  |
| A125 | VSNSTCRFFVCKCVE | 1 | 0 |  | FKA**(DRYKSHGKGY)**NW | **DRYKSHGKGY** | 8 | 15 |  |
| A126 | TC**(RFFVCKCVER)**RAE | 33 | 0 | **A*33:03 (A03)** | FKADR**(YKSHGKGYNW)** | **YKSHGKGYNW** | **480** | **170** |  |
| A127 | FVCKCVERRAEVTSN | 1 | 0 |  | **(FKADRYKSH)**GKGYNW | **FKADRYKSH** | 0 | 0 |  |
| A128 | CVERRAEVTSNNEVV | 0 | 0 |  | F**(KADRYKSHGK)**GYNW | **KADRYKSHGK** | 8 | 5 |  |
| A129 | RAEVTSNNEVVVKEE | 1 | 1 |  | FKADRY**(KSHGKGYNW)** | **KSHGKGYNW** | **975** | **303** |  |
| A130 | TSNNEVVVKEEYKDE | 1 | 9 |  | FKA**(DRYKSHGK)**GYNW | **DRYKSHGK** | 8 | **35** |  |
|  | | | | | FK**(ADRYKSHGK)**GYNW | **ADRYKSHGK** | 5 | **30** |  |
|  |  |  |  |  | **A98** |  |  |  |  |
|  |  |  |  |  | RYKSHGKGYNWGNYN |  | **365** | 8 |  |
|  |  |  |  |  | RYKS**(HGKGYNWGNY)**N | **HGKGYNWGNY** | 0 | 0 |  |
|  |  |  |  |  |  |  |  |  |  |
|  |  |  |  |  | **A126** |  |  |  |  |
|  |  |  |  |  | TCRFFVCKCVERRAE |  | **48** | **28** |  |
|  |  |  |  |  | TC**(RFFVCKCVER)**RAE | **RFFVCKCVER** | **160** | **113** |  |

PBMCs were collected post-ChAd63/pre-CHMI. **(A)** All 15mer peptides within Ap4 and Ap10 were tested in FluoroSpot assays. **(B)** All 15mer peptides within Ap8 were tested in FluoroSpot assays. Positive activities are shown in bold and predicted minimal epitopes within 15mers are shown in bold with parenthesis and underlined (**C**) Each predicted epitope shown was synthesized and tested.
