# Supplementary material for: Human responses to the DNA prime/chimpanzee adenovirus (ChAd63) boost vaccine identify CSP, AMA1 and TRAP MHC Class I-restricted epitopes
Source: PLoS One. 2025 Feb 13;20(2):e0318098. doi: 10.1371/journal.pone.0318098 (PMC11825025; doi:10.1371/journal.pone.0318098)
Supplement: S10 Table — (DOCX) [file pone.0318098.s010.docx]

**S10 Table. Cohort CAT: FluoroSpot IFN-γ and GzB responses for non-protected participant v33 (HLA A24/A03, B07/B58) to 3D7 TRAP TD3 peptide pool and 15mer peptides containing predicted epitopes**

| **Response to TD3 subpools and 15mer components** | | | | |
| --- | --- | --- | --- | --- |
| **Pool/ 15mer** | **15mer Sequence** | **IFN-γ sfc/m** | **GzB sfc/m** | **HLA restriction/ST of predicted epitope** |
| **TD3** |  | **603** | **88** |  |
| SS-51 | FLVGCHPSDGKCNLY | 8 | 0 |  |
| SS-52 | CHPSDGKCNLYADSA | 0 | 0 |  |
| SS-53 | DG**(KCNLYADSAW)**ENV | **30** | 25 | **B*58:01 (B58)** |
| SS-54 | NLYADSAWENVKNVI | 0 | 5 |  |
| SS-55 | D**(SAWENVKNV)**IGPFM | **48** | **60** | **B*51:01 (B07)** |
| SS-56 | ENVKNVIGPFMKAVC | **30** | 0 |  |
| SS-57 | NVIGPFMKAVCVEVE | 13 | 0 |  |
| SS-58 | PFMKAVCVEVEKTAS | 8 | 5 |  |
| SS-59 | AVCVEVEKTASCGVW | 10 | 8 |  |
| SS-60 | EVEKT**(ASCGVWDEW)**S | **1095** | **173** | **B*58:01 (B58)** |
| SS-61 | T**(ASCGVWDEW)**SPCSV | **135** | **30** | **B*58:01 (B58)** |
| SS-62 | GVWDEWSPCSVTCGK | 3 | 0 |  |
| SS-63 | EWSPCSVTCGKGTRS | 0 | 0 |  |
| SS-64 | CSVTCGKGTRSRKRE | 0 | 0 |  |
| SS-65 | CGKGTRSRKREILHE | 0 | 0 |  |
| SS-66 | TRSRKREILHEGCTS | 0 | 0 |  |
| SS-67 | KREILHEGCTSELQE | 0 | 3 |  |
| SS-68 | LHEGCTSELQEQCEE | 0 | 0 |  |
| SS-69 | CTSELQEQCEEERCL | 0 | 0 |  |
| SS-70 | LQEQCEEERCLPKRE | 0 | 5 |  |
| SS-71 | CEEERCLPKREPLDV | 0 | 0 |  |
| SS-72 | RCLPKREPLDVPDEP | 0 | 0 |  |
| SS-73 | KREPLDVPDEPEDDQ | 0 | 0 |  |
| SS-74 | LDVPDEPEDDQPRPR | 0 | 0 |  |
| SS-75 | DEPEDDQPRPRGDNF | 3 | 0 |  |

PBMCs were collected post-ChAd63/pre-CHMI. All 15mer peptides within TD3 were tested in FluoroSpot assays. Positive 15mers activities are shown in bold. Predicted minimal epitopes within 15mers are in bold with parenthesis and underlined.
