# Supplementary material for: Human responses to the DNA prime/chimpanzee adenovirus (ChAd63) boost vaccine identify CSP, AMA1 and TRAP MHC Class I-restricted epitopes
Source: PLoS One. 2025 Feb 13;20(2):e0318098. doi: 10.1371/journal.pone.0318098 (PMC11825025; doi:10.1371/journal.pone.0318098)
Supplement: S11 Table — (DOCX) [file pone.0318098.s011.docx]

**S11 Table. Cohort CAT: FluoroSpot responses for non-protected participant v35 HLA A02/A02, B27/B58 to AMA1 Ap8 peptide pool, 15mer peptides, and synthesized predicted epitopes**

| **A. Response to sub pool and 15mers components** | | | | |  | **B. Response to positive 15mer and predicted epitopes** | | | |
| --- | --- | --- | --- | --- | --- | --- | --- | --- | --- |
| **Pool/**  **15mer** | **15mer Sequence** | **IFN-γ**  **sfc/m** | **GzB**  **Sfc/m** | **HLA restriction/ST of predicted epitope** |  | **15mer Sequence** | **Epitope** | **IFN-γ**  **sfc/m** | **GzB**  **Sfc/m** |
| **Ap8** |  | **325** | 25 |  |  | **A97** |  |  |  |
| A92 | EGFKNKNASMIKSAF | 4 | 8 |  |  | FKADRYKSHGKGYNW |  | **560** | **133** |
| A93 | NKNASMIKSAFLPTG | 1 | 3 |  |  | FKAD**(RYKSHGKGY)**NW | **RYKSHGKGY** | 18 | 0 |
| A94 | SMIKSAFLPTGAFKA | 8 | 3 |  |  | FKA**(DRYKSHGKGY)**NW | **DRYKSHGKGY** | 0 | 0 |
| A95 | SAFLPTGAFKADRYK | 1 | 0 |  |  | FKADR**(YKSHGKGYNW)** | **YKSHGKGYNW** | **135** | **85** |
| A96 | PTGAFKADRYKSHGK | 1 | 3 |  |  | **(FKADRYKSH)**GKGYNW | **FKADRYKSH** | 8 | 20 |
| A97 | **(FKADRYKSHGKGYNW)** | **470** | **53** | **B*58:01 (B58)** |  | F**(KADRYKSHGK)**GYNW | **KADRYKSHGK** | 0 | 0 |
| A98 | RYKSHGKGYNWGNYN | 30 | 8 |  |  | FKADRY**(KSHGKGYNW)** | **KSHGKGYNW** | **393** | 30 |
| A99 | HGKGYNWGNYNTETQ | 0 | 0 |  |  | FKA**(DRYKSHG)**KGYNW | **DRYKSHG** | 3 | 13 |
| A100 | YNWGNYNTETQKCEI | 1 | 13 |  |  | FK**(ADRYKSHGK)**GYNW | **ADRYKSHGK** | 0 | 0 |
| A101 | NYNTETQKCEIFNVK | 0 | 3 |  |  |  | | | |
| A102 | ETQKCEIFNVKPTCL | 0 | 0 |  |  |  |  |  |  |
| A103 | CEIFNVKPTCLINNS | 8 | 0 |  |  |  |  |  |  |
| A104 | NVKPTCLINNSSYIA | 0 | 0 |  |  |  |  |  |  |

PBMCs were collected post-ChAd63/pre-CHMI. All 15mer peptides within Ap8 were tested in FluoroSpot assays. **(A)** Positive 15mers activities are shown in bold. Predicted minimal epitopes within 15mers are shown in bold with parenthesis and underlined.

**(B)** Predicted minimal epitopes within the positive 15mer A97 were synthesized and tested. Positive activities are shown in bold.
