# Supplementary material for: Human responses to the DNA prime/chimpanzee adenovirus (ChAd63) boost vaccine identify CSP, AMA1 and TRAP MHC Class I-restricted epitopes
Source: PLoS One. 2025 Feb 13;20(2):e0318098. doi: 10.1371/journal.pone.0318098 (PMC11825025; doi:10.1371/journal.pone.0318098)
Supplement: S12 Table — (DOCX) [file pone.0318098.s012.docx]

**S12 Table. Cohort CAT: FluoroSpot responses for non-protected participant v35 HLA A02/A02, B27/B58 to TRAP TD1 and TD3 peptide pools, 15mer peptides, and predicted epitopes**

| **A. Response to subpools and 15mer components** | | | | |  | **B. Response to subpools and 15mer components** | | | | |
| --- | --- | --- | --- | --- | --- | --- | --- | --- | --- | --- |
| **TD1**  **15mer** | **15mer Sequence** | **IFN-γ**  **sfc/m** | **GzB**  **sfc/m** | **HLA restriction/ST of predicted epitope** |  | **TD3**  **15mer** | **15mer Sequence** | **IFN-γ**  **sfc/m** | **GzB**  **sfc/m** | **HLA restriction/ST of predicted epitope** |
| **TD1** |  | **63** | **53** |  |  | **TD3** |  | **203** | 30 |  |
| SS-1 | MNHLGNVKYLVIVFL | 0 | 3 | A*02:01 (A02)* |  | SS-51 | FLVGCHPSDGKCNLY | 0 | 0 |  |
| SS-2 | GNVKYLVIVFLIFFD | 0 | 10 |  |  | SS-52 | CHPSDGKCNLYADSA | 0 | 10 |  |
| SS-3 | YLVIVFLIFFDLFLV | 0 | 7 |  |  | SS-53 | DGKCNLYADSAWENV | 7 | 13 | B*58:01 (B58)* |
| SS-4 | VFLIFFDLFLVNGRD | 0 | 7 | A*02:01 (A02)* |  | SS-54 | NLYADSAWENVKNVI | 0 | 0 |  |
| SS-5 | FFDLFLVNGRDVQNN | 3 | 10 |  |  | **SS-55** | D(**SAWENVKNV)**IGPFM | **47** | **257** | **A*02:06 (A02)** |
| SS-6 | FLVNGRDVQNNIVDE | 0 | 20 |  |  | SS-56 | ENVKNVIGPFMKAVC | 0 | 0 |  |
| SS-7 | GRDVQNNIVDEIKYR | 7 | 13 |  |  | SS-57 | NVIGPFMKAVCVEVE | 3 | 0 |  |
| SS-8 | QNNIVDEIKYREEVC | 0 | 17 |  |  | SS-58 | PFMKAVCVEVEKTAS | 0 | 3 |  |
| SS-9 | VDEIKYREEVCNDEV | 0 | 0 |  |  | SS-59 | AVCVEVEKTASCGVW | **47** | **77** |  |
| SS-10 | KYREEVCNDEVDLYL | 10 | 10 |  |  | **SS-60** | EVEKT**(ASCGVWDEW)**S | **197** | 13 | **B*58:01 (B58)** |
| SS-11 | EVCNDEVDLYLLMDC | 7 | 0 |  |  | **SS-61** | T**(ASCGVWDEW)**SPCSV | **70** | 13 | **B*58:01 (B58)** |
| SS-12 | DEVDLYLLMDCSGSI | 0 | 27 | A*02:01 (A02)* |  | SS-62 | GVWDEWSPCSVTCGK | 0 | 0 |  |
| **SS-13** | LY**(LLMDCSGSI)**RRHN | 20 | **43** | **A*02:01 (A02)** |  | SS-63 | EWSPCSVTCGKGTRS | 0 | 3 |  |
| SS-14 | MDCSGSIRRHNWVNH | 0 | 3 |  |  | SS-64 | CSVTCGKGTRSRKRE | 0 | 0 |  |
| SS-15 | GSIRRHNWVNHAVPL | 3 | 7 |  |  | SS-65 | CGKGTRSRKREILHE | 7 | 0 |  |
| SS-16 | RHNWVNHAVPLAMKL | 0 | 0 |  |  | SS-66 | TRSRKREILHEGCTS | 0 | 0 |  |
| SS-17 | VNHAVPLAMKLIQQL | 3 | 10 |  |  | SS-67 | KREILHEGCTSELQE | 0 | 7 |  |
| SS-18 | VPLAMKLIQQLNLND | 0 | 3 |  |  | SS-68 | LHEGCTSELQEQCEE | 0 | 0 |  |
| SS-19 | MKLIQQLNLNDNAIH | 0 | 0 |  |  | SS-69 | CTSELQEQCEEERCL | 0 | 3 |  |
| SS-20 | QQLNLNDNAIHLYAS | 0 | 0 | A*02:01 (A02)* |  | SS-70 | LQEQCEEERCLPKRE | 0 | 0 |  |
| SS-21 | LNDNAIHLYASVFSN | 0 | 7 |  |  | SS-71 | CEEERCLPKREPLDV | 0 | 0 |  |
| **SS-22** | A**(IHLYASVFS)**NNARE | **57** | **70** | **B*39.05 (B27)** |  | SS-72 | RCLPKREPLDVPDEP | 0 | 3 |  |
| **SS-23** | YASV**(FSNNAREII)**RL | **60** | **67** | **B*58:01 (B58)** |  | SS-73 | KREPLDVPDEPEDDQ | 0 | 0 |  |
| SS-24 | FSNNAREIIRLHSDA | 0 | 3 |  |  | SS-74 | LDVPDEPEDDQPRPR | 0 | 3 |  |
| SS-25 | AREIIRLHSDASKNK | 0 | 3 |  |  | SS-75 | DEPEDDQPRPRGDNF | 0 | 7 |  |

PBMCs were collected post-ChAd63/pre-CHMI. All 15mer peptides within (**A**) TD1 and (**B**) TD3 were tested in FluoroSpot assays. Positive 15mers activities are shown in bold. Predicted epitopes within 15mers are shown in bold with parenthesis and underlined.

Amino acids in the predicted epitopes that vary between 3D7 and T9/96 are shown in red. *Epitopes recognized by protected v24 that also expresses HLA A02 and non-protected v33 that also expresses HLA B58 are shown as non-bold.
