# Supplementary material for: Human responses to the DNA prime/chimpanzee adenovirus (ChAd63) boost vaccine identify CSP, AMA1 and TRAP MHC Class I-restricted epitopes
Source: PLoS One. 2025 Feb 13;20(2):e0318098. doi: 10.1371/journal.pone.0318098 (PMC11825025; doi:10.1371/journal.pone.0318098)
Supplement: S13 Table — (DOCX) [file pone.0318098.s013.docx]

**S13 Table. Summary of predicted but not confirmed CSP, AMA1 and TRAP MHC Class I-restricted epitopes**

| **CSP subpool** | **CSP Peptide** | **15mer Sequence** | **HLA-restriction** | **Response** |
| --- | --- | --- | --- | --- |
| **Cp5** | C45 | NNNNE**(EPSDKHIKEY)** | B*35:01 (B07) | IFN-γ |
|  |  | NNNNEE**(PSDKHIKEY)** | A*01:01 (A01) | IFN-γ |
| **AMA1 subpool** | **AMA1 Peptide** | **15mer Sequence** | **HLA-restriction** | **Response** |
| **Ap1** | A7 | Ac-QNYWE**(HPYQNSDVY)**R | B*35:01 (B07) | IFN-γ |
|  | A8 | E**(HPYQNSDVY)**RPINE | B*35:01 (B07) | IFN-γ |
| **Ap8** | A103 | **(CEIFNVKPT)**CLINNS | B*45:01 (B44) | GzB |
| **TRAP subpool** | **TRAP Peptide** | **15mer Sequence** | **HLA-restriction** | **Response** |
| **TD1** | SS-1 | MN**(HLGNVKYLV)**IVFL | A*02:01 (A02) | IFN-γ/GzB |
|  | SS-2 | GNV**(KYLVIVFLI)**FFD | A*32:01 (A01) | GzB |
|  | SS-4 | V**(FLIFFDLFLV)**NGRD | A*02:01 (A02) | GzB |
|  | SS-5 | FFDL**(FLVNGRDVQ)**NN | B*35:01 (B07) | GzB |
|  | SS-8 | QNNIV**(DEIKYREEV)**C | B*44:03 (B44) | IFN-γ/GzB |
|  | SS-9 | V**(DEIKYREEVC)**NDEV | B*44:03 (B44) | IFN-γ/GzB |
|  | SS-10 | KYR**(EEVCNDEVDL)**YL | B*44:03 (B44) | GzB |
|  | SS-11 | E**(VCNDEVDLY)**LLMDC | B*35:01 (B07) | GzB |
|  | SS-12 | DEVDLY**(LLMDCSGSI)** | A*02:01 (A02) | GzB |
|  | SS-13 | LY**(LLMDCSGSI)**RRHN | A*02:01 (A02) | GzB |
|  | SS-14 | MDCS**(GSIRRHNW)**VNH | A*32:01 (A01) | GzB |
|  | SS-15 | GSIR**(RHNWVNHAV)**PL | A*32:01 (A01) | GzB |
|  | SS-16 | RHNWVN**(HAVPLAMKL)** | B*35:01 (B07) | GzB |
|  | SS-17 | VN**(HAVPLAMK)**LIQQL | B*35:01 (B07) | GzB |
|  | SS-18 | VP**(LAMKLIQQL)**NLND | B*35:01 (B07) | GzB |
|  | SS-20 | QQL**(NLNDNAIHL)**YAS | A*02:01 (A02) | GzB |
|  | SS-21 | LND**(NAIHLYASVF)**SN | B*35:01 (B07) | IFN-γ/GzB |
|  | SS-22 | **(AIHLYASVF)**SNNARE | B*35:01 (B07); B*39.05 (B27) | IFN-γ/GzB |
|  | SS-23 | **(YASVFSNNA)**REIIRL | B*35:01 (B07); B*58:01 (B58) | GzB; IFN-γ; IFN-γ/GzB |
|  | SS-24 | FSNN**(AREIIRLHSDA)** | B*44:03 (B44) | GzB |
| **TD2** | SS-28 | KNKEK**(ALIIIKSL)**LS | A*02:01 (A02) | IFN-γ/GzB |
|  | SS-30 | IIK**(SLLSTNLP)**YGKT | B*35:01 (B07) | IFN-γ/GzB; IFN-γ |
|  | SS-32 | NLPYG**(KTNLTDALL)**Q | A*32:01 (A01) | IFN-γ |
|  | SS-36 | RK**(HLNDRINRE)**NANQ | A*02:01 (A02) | GzB; IFN-γ/GzB |
|  | SS-38 | N**(RENANQLVV)**ILTDG | B*44:03 (B44) | IFN-γ/GzB |
|  |  | NRE**(NANQLVVIL)**TDG | B*35:01 (B07) | IFN-γ/GzB |
|  | SS-42 | P**(DSIQDSLKE)**SRKLS | B*35:01 (B07) | GzB |
|  | SS-43 | QDSL**(KESRKLSDRG)**V | B*44:03 (B44) | IFN-γ/GzB |
|  | SS-44 | KESR**(KLSDRGVKI)**AV | A*32:01 (A01) | GzB |
|  | SS-46 | RGVKI**(AVFGIGQGI)**N | A*32:01 (A01) | GzB |
|  | SS-49 | GIN**(VAFNRFLVG)**CHP | B*42:02 (B07) | GzB |
|  | SS-50 | AFN**(RFLVGCHPS)**DGK | A*30:01 (A01A03) | GzB |
| **TD3** | SS-53 | DG**(KCNLYADSAW)**ENV | B*58:01 (B58) | IFN-γ |
|  | SS-55 | D**(SAWENVKNV)**IGPFM | B*51:01 (B07); A*02:06 (A02) | IFN-γ/GzB |
|  | SS-60 | EVEKT**(ASCGVWDEW)**S | B*58:01 (B58) | IFN-γ/GzB; IFN-γ |
| **TD5** | SS-104 | D**(RYIPYSPLS)**PKVLD | A*30:01 (A01A03) | IFN-γ |

All predicted TRAP epitopes within TD1, TD2, TD3 and TD5. Predicted minimal epitopes within 15mers are shown in bold within parenthesis and underlined. Amino acids in the predicted epitopes that vary between 3D7, and T9/96 are shown in red. IFN-γ and GzB responses for each epitope are shown once when responses are similar for individual participants or separated by a semicolon when responses differed among more than one individual participants.
