# Supplementary material for: Human responses to the DNA prime/chimpanzee adenovirus (ChAd63) boost vaccine identify CSP, AMA1 and TRAP MHC Class I-restricted epitopes
Source: PLoS One. 2025 Feb 13;20(2):e0318098. doi: 10.1371/journal.pone.0318098 (PMC11825025; doi:10.1371/journal.pone.0318098)
Supplement: Attachment — Submitted filename: Response to Reviewers.docx [file pone.0318098.s014.docx]

PONE-D-24-16201

The DNA prime/chimpanzee adenovirus (ChAd63) boost vaccine confers sterile immunity associated with CSP, AMA1 and TRAP MHC Class I-restricted epitopes

**Response to Reviewers** (line numbers refer to the revised manuscript without tracked changes)

**REVIEWER #1**

The authors need an opening statement to orientate the readers to malaria and the challenges phased in control, hence the need for vaccines.

- Response: We agree, and we have added a paragraph to the Introduction lines 86-98.

Lines 163: Immunological samples: what is the justification for collection of PBMC on day 27?

- Response: This time point is 27 days after the ChAd63 boost, and 5 or 6 days before CHMI (lines 204-205).

Line 187, Fresh or cryopreserved PBMCs we expect some differences in results, how was this addressed, especially in the results presented?

- Response: We have previously shown that ELISpot activities of fresh or cryopreserved PBMC differ in magnitude but not antigen-specificity and we cite two publications [19,22]. We also state “Since fresh PBMC were used for initial evaluations with sub-pools, and cryopreserved PMBC were used for all 15mer and epitope evaluations, we consider that both evaluations are consistent.” (lines 208-212).

The authors mention the use of Mann-Whitney U test in their methods, However, results/ discussion no mention is made in relation to the Mann-Whitney test results.

- Response: This was included in an earlier draft and should have been deleted.

Discussion: pervious studies using huAd5 in malaria, eg Marvin J Saklar, were not included how does this overall compare to other manuscripts?

- Response: This vaccine described here was a DNA prime/ChAd63 boost regimen (Sklar et al reference #16, and we consistently refer to our earlier study that used DNA prime/HuAd5 boost (reference #10). We also cite a previous vaccine study using HuAd5 alone (lines 599-601) reference #6. We respectfully suggest that a longer review of HuAd5 malaria vaccines is not relevant to this report.

**REVIEWER #2**

But the authors should explain how they can justify the relationship between the immunogenicity of peptides and protection. (It is possible that other immune mechanisms like humoral immunity are responsible for the protection.)

- Response: This is an entirely reasonable and relevant comment, and we have clarified the manuscript to ensure that we cite our published analyses of humoral responses that were not correlated with VE (line 126; lines 142-143).
- We have modified the title of this study to say we identified epitopes and in the text, we discuss whether we can say that these are associated with protection.

Similarly, even if CD8 T cell activating peptides are included in the TRAP sequence, its role in protection remains still unclear.

- Response: Yes, we completely agree. We have added a subsection that states that we cannot conclude that TRAP epitopes are associated with protection (lines 620-622)

The authors predicted and confirmed MHC Class-I restricted epitopes which stimulate T cell and induce IFN-g and GzB production. But authors described “some non-protected individuals recognized HLA-matched protective minimal epitopes. (Line 53-54). How do authors think the reliability of the prediction?

Response: There are really two questions here. Firstly, how reliable is the NetMHCpan-4.0 algorithm in predicting Class I-restricted epitopes that recall responses using the FluoroSpot assay? The algorithm is not designed to predict protective epitopes, only to predict epitopes that would recall responses. 10 different predicted epitopes were synthesized, and seven predicted epitopes recalled FluoroSpot response, whereas 3 predicted epitopes did not recall responses. This has been added (lines 629-635).

The second question is how specific to protection are any of these epitopes. Given the small sample size it is difficult to determine whether each epitope only recalled responses from protected or non-protected participants (lines 636-643). However, this analysis also suggests that co-expressed HLA alleles may influence protection (lines 519-545 and 577-608).

We thank Reviewer #2 for raising these questions which strengthen the manuscript.

Sample number is limited, and it cannot cover HLA various alleles. How do authors think the universality of your findings?

- Response: A major factor that will influence coverage of a genetically diverse population is the promiscuity of Class I epitopes. We show here an example of an epitope restricted by two different HLA alleles and promiscuity of the validated epitopes is included in Table 7.

According to the 1-3, their title “The DNA prime/chimpanzee adenovirus (ChAd63) boost vaccine confers sterile immunity associated with CSP, AMA1 and TRAP MHC Class I-restricted epitopes” seems too strong.

- Response: We have better defined our meaning of associated with protection as defined by FluoroSpot assays but not as protective epitopes that elicit protection to malaria after epitope immunization (lines 674-690). Therefore, we suggest that our title using “associated” is appropriate.

I could not find the legend for Figure 1. Therefore, it is difficult to provide a final evaluation of this manuscript with the information provided.

- Response: This arose during submission of the original manuscript and has now been corrected.

More detailed information of CHMI and definition of protected and non-protected should be provided in the methods part (even I could see this study following Reference 17).

- Response: The details of CHMI and definition of VE are fully described in the cited publication of the clinical trial (reference #16) and is described in lines 118-124.

**REVIEWER #3**

I find this manuscript problematic to read. On the one hand, it abounds in technical information that requires exhaustive knowledge of the subject. On the other hand, the structure of the text is not too appropriate. Several sections allude to the supplement tables, and it is unknown if they are part of the main text or the legend of the tables.

- Response: We have seriously tried to edit this manuscript to make it easier to read, and especially by including summaries of findings in each cohort in the form of bullet points. We have better emphasized the main finding that the NetMHCpan algorithm predicted a series of Class I-restricted epitopes that we verified in FluoroSpot assays that identified 7 epitopes that could form the foundation of a multi-epitope vaccine. Further, we have simplified the Tables to better show the activities of synthesized epitopes relative to their parent 15mers and subpools. We also state that the complete data is shown in the Supplementary Tables.

I view the statistical analysis as absent. The statistical analysis section in the manuscript has only one sentence. It establishes that the study has considered the Mann-Whitney test, but it no longer appears in any presentation or discussion of results. I can't quickly identify the comparisons made with the Mann-Whitney test. Besides, if multiple comparisons are needed (I think so), the family-wise error should be corrected appropriately.

- Response: As stated for Reviewer #1, the Mann-Whitney test was in an earlier draft but was not used here. The aim of this analysis was to only identify vaccine antigen epitopes that were associated with protection, and not to quantitatively compare responses as these were reported in our first publication reference #16. Therefore, this is a qualitative analysis of antigen epitope identification.

Another concern is the sample size in each group. On the one hand, the sample sizes are small, and the authors emphasize many individual particularities in several subjects, so I wonder about the validity of the inferences.

- We totally agree that this is a valid concern. We were constrained by the number of participants in the original DNA/ChAd63 clinical trial (lines 191-195). The second constraint was the availability of cryopreserved PBMCs that is described under Limitations (lines 287-288; 562-564; 636-637; 777-782). The FluoroSpot assays are intensive studies and require significant numbers of PBMCs that further restrict the numbers of participants that can be utilized. We and others have previously used similar integrated methods to identify relevant epitopes that could be used in further studies such as better-focused immunological assays or as a foundation of a multi-epitope vaccine.

On the other hand, my view is that the paper does not state a clear relationship between the two experimental groups and the protected/unprotected subjects.

- We clearly define the two cohorts CA and CAT (lines 104-107) and the outcome of CHMI challenge (lines 118-121). We also state that PBMCs were taken pre-CHMI and represent only vaccine-induced responses (lines 140-141; 204-205).
